# Leerklimaat perceptie van aios en supervisors binnen de vernieuwde medische vervolgopleiding arts Maatschappij + Gezondheid

**DOI:** 10.1007/s12508-022-00367-6

**Published:** 2022-11-08

**Authors:** Tessa N. de Wit, Nadieh Taks, Angarath I. van der Zee-van den Berg, Sheda Sadrzadeh

**Affiliations:** Netherlands School of Public and Occupational Health, Utrecht, Nederland

**Keywords:** leerklimaat, sociale geneeskunde, arts M + G, D‑RECT, medische vervolgopleiding, Learning climate, Public health, D‑RECT, Postgraduate medical education

## Abstract

**Inleiding:**

Het doel van dit onderzoek was het evalueren van de ervaren kwaliteit van het leerklimaat door artsen in opleiding (aios) binnen de medische vervolgopleiding arts Maatschappij + Gezondheid (M + G) en het vergelijken van de percepties van aios en supervisors.

**Methode:**

Aios van vijf profielen arts M + G, die in 2019 en later met hun opleiding zijn begonnen, en supervisors die betrokken zijn bij het opleidingsprogramma werden uitgenodigd om een online vragenlijst in te vullen op basis van een aangepaste versie van de D‑RECT-vragenlijst. De antwoorden van aios en supervisors van dezelfde opleidingsinstelling en hetzelfde profiel werden gematcht om de waargenomen kwaliteit van het leerklimaat te vergelijken.

**Resultaten:**

Honderdveertien aios reageerden (respons 50,9 %). De algemene beoordeling van het leerklimaat door de aios gaf een gemiddelde score van 4,19 op een vijfpuntsschaal. Achtendertig supervisor-aios-matches werden gevormd. Er waren geen noemenswaardige verschillen in de beleving van aios en supervisors.

**Conclusie:**

De algemene beoordeling van het leerklimaat door de aios was positief. De percepties van het leerklimaat door supervisors en aios zijn vergelijkbaar. Onze aangepaste versie van D‑RECT lijkt geschikt om het leerklimaat van de medische vervolgopleiding arts M + G te evalueren. Herhaling van het onderzoek is nodig om het leerklimaat op lokaal niveau te beoordelen en onze bevindingen te bevestigen. Verdere aanpassing en validering van de vragenlijst is wenselijk voor een betere weergave van de werk- en leeromgeving van de arts M + G.

**Digitaal aanvullende content:**

De online versie van dit artikel (10.1007/s12508-022-00367-6) bevat aanvullend materiaal, toegankelijk voor daartoe geautoriseerde gebruikers.

## Abstract (en)

**Introduction:**

The purpose of this study was to evaluate the perceived quality of the learning climate by public health residents in the Netherlands and compare residents’ and supervisors’ perceptions.

**Methods:**

Residents of five public health subfields, who started their residency programs in 2019 and onwards, as well as supervisors involved in the residency program, were invited to complete a web-based survey based on an adapted version of the D‑RECT questionnaire. Answers of residents and supervisors of the same training site and public health subfield were matched to compare perceived quality of the learning climate.

**Results:**

One hundred fourteen residents responded (response rate 50.9%). Residents’ overall assessment of the learning climate showed a mean score of 4.19 on a 5-point-scale. Thirty-eighth supervisor-resident matches were formed. There were no notable differences in the perception of residents and supervisors.

**Conclusion:**

Residents’ overall assessment of the learning climate was positive. Supervisors and residents’ perception of learning climate is equal. Our adapted version of D‑RECT seems to be suitable to evaluate the learning climate for public health residency programs in the Netherlands. Further research is necessary to validate our questionnaire and to confirm our findings.

## Inleiding

### Leerklimaat

Leerklimaat is een sleutelcomponent van de kwaliteit van de medische vervolgopleiding en kan worden gedefinieerd als de sociale, emotionele en fysieke omstandigheden waaronder de arts in opleiding (aios) kennis verwerft [1–3]. Het omvat aspecten als algemeen heersende werkhouding en sfeer van een instelling [4]. Het leerklimaat is ook nauw gerelateerd aan leer- en klinische uitkomsten [5–7]. Bovendien beïnvloedt het leerklimaat de perceptie van de aios van zijn eigen competenties [8]. De ervaren kwaliteit van het leerklimaat hangt ook omgekeerd samen met burn-out onder aios [9]. Er is een positieve samenhang met een betere kwaliteit van leven, meer balans tussen werk en privé, en minder symptomen van emotionele uitputting en depersonalisatie [10]. Bij werkplekleren is een veilig leerklimaat een van de belangrijkste factoren voor effectief leren [11, 12], en een voorwaarde om optimaal te kunnen profiteren van leermomenten [13]. Vanwege de genoemde grote impact van het leerklimaat op de prestaties en het welzijn van aios en de centrale rol van aios in het leerproces is het belangrijk om de kwaliteit van het leerklimaat regelmatig te evalueren en te verbeteren [14, 15].

### Perceptie van aios versus die van supervisors

Bij het evalueren van het leerklimaat kan het van toegevoegde waarde zijn om ook het perspectief van de supervisors mee te nemen. Uit eerder onderzoek blijkt dat werken in dezelfde omgeving, maar met een andere rol, kan leiden tot een andere beoordeling van het leerklimaat. Medische studenten en chirurgische arts-assistenten waren het eens over universele elementen die bijdragen aan een optimaal leerklimaat voor alle leerlingen, maar verschilden van mening over elementen die meer verband houden met de verschillen in hun opleidingsniveau [16]. Percepties van supervisors en aios lijken ook bij sommige onderwerpen uiteen te lopen, waaronder de kwaliteit van het onderwijs en feedback, patiëntveiligheidscultuur en ideeën over wanneer de superviserende arts op de hoogte moet worden gesteld [17–19]. Supervisors en aios noemen ook andere sterke punten en kansen voor verbetering in een medische vervolgopleiding [20]. Voor het optimaliseren van het leerklimaat is het daarom belangrijk om het perspectief van alle stakeholders mee te nemen.

### Opleiding arts Maatschappij + Gezondheid

In 2019 is het curriculum voor de aios in opleiding tot arts Maatschappij + Gezondheid (M + G) ingrijpend gewijzigd. Deze aios zijn nu in dienst bij één landelijke werkgever, waardoor het mogelijk is om de praktijkleerperiodes en stages bij verschillende opleidingsinstellingen te volgen. Voorheen volgden de aios hun hele opleiding op de werkplek waar ze voor aanvang van hun opleiding al werkzaam waren. Zo’n grote curriculumverandering kan impact hebben op het leerklimaat.

Het werkveld van de arts M + G is onderverdeeld in profielen [21]. De Netherlands School of Public and Occupational Health (NSPOH) biedt een tweejarige opleiding aan op alle deelgebieden van de arts M + G, leidend tot een KNMG-registratie als profielarts (eerste fase) en een daaropvolgende tweejarige specialisatie die leidt tot volledige registratie als geneeskundig specialist (tweede fase). Dit onderzoek richtte zich op de volgende eerstefaseprofielen: Jeugdgezondheidszorg (JGZ), Forensische Geneeskunde (FG), Infectieziektebestrijding (IZB), Tuberculosebestrijding (TBC) en Medische Milieukunde (MMK). De laatste drie volgen gedeeltelijk een gecombineerd curriculum, hebben een grotendeels gelijkaardige werkomgeving en worden in dit artikel dan ook gezamenlijk ITM genoemd. Hoewel niet volledig zullen we voor de leesbaarheid de vijf onderzochte profielen samen aanduiden als arts M + G. De tweejarige opleiding voor JGZ wordt ook aangeboden door een tweede opleidingsinstituut, namelijk TNO Child Health.

De aios volgen een praktijkgerichte opleiding ondersteund door cursorisch onderwijs, dat door de opleidingsinstituten op centrale locaties verzorgd wordt. Op fulltimebasis behelst de praktijkopleiding twee periodes van in totaal vijftien maanden praktijkleren in het eigen profiel, aangevuld door drie keer drie maanden externe stages. Werkplekleren in het eigen profiel vindt plaats bij opleidingsinstellingen door heel Nederland, zoals GGD’en, het Rijksinstituut voor Volksgezondheid en Milieu (RIVM) en Centra voor Jeugd en Gezin. Tijdens een praktijkperiode wordt iedere aios gekoppeld aan een praktijkopleider op de werkvloer. De praktijkopleider fungeert als mentor en bewaakt de voortgang van de praktijkopleiding. Daarnaast zijn andere artsen werkzaam in dezelfde instelling vaak betrokken bij het superviseren en begeleiden van aios bij werkplekleren. Instituutopleiders verbonden aan de opleidingsinstituten NSPOH en TNO bewaken de voortgang van de aios en geven vorm aan de opleiding, rekening houdend met de landelijke opleidingskaders.

De NSPOH evalueert het curriculum volgens een cyclisch proces zoals gedefinieerd door de kwaliteitsrichtlijnen van de Koepel Artsen Maatschappij + Gezondheid [22]. Tot nu toe was het leerklimaat op de werkplek echter geen expliciet onderdeel van het evaluatieproces. Omdat het curriculum een grote verandering heeft ondergaan en vanwege de impact van het leerklimaat op de prestaties en het welzijn van de aios, is het waardevol om het leerklimaat in de nieuwe situatie te evalueren. Hierbij zijn het perspectief van de aios en dat van de supervisor opgenomen omdat zij belangrijke belanghebbenden zijn.

Het doel van dit onderzoek is daarom om de ervaren kwaliteit van het leerklimaat door aios M + G binnen de opleidingsinstelling op nationaal niveau per profiel te evalueren. Ons tweede doel is om het perspectief ten aanzien van het leerklimaat van de aios en supervisors met elkaar te vergelijken. Dit onderzoek richt zich op werkplekleren in het kader van het eigen profiel, het leerproces tijdens de externe stages blijft buiten beschouwing. Voor zover bij ons bekend, is ons onderzoek het eerste naar het leerklimaat binnen de arts M + G-profielopleiding vanuit het perspectief van de aios en supervisor.

## Methode

### Setting en onderzoekspopulatie

In juni 2021 zijn alle 224 Nederlandse aios M + G (profiel JGZ 137, IZB 44, TBC 2, MMK 8 en FG 33) die in 2019 en later met hun opleiding zijn begonnen, en hun 195 praktijkopleiders (128 JGZ, 33 IZB, 3 TBC, 7 MMK en 18 FG) gevraagd om deel te nemen aan ons onderzoek. Aios en praktijkopleiders zijn direct per e‑mail uitgenodigd, gebruikmakend van de inschrijfgegevens van de aios en praktijkopleiders van de NSPOH en TNO. Aios werd gevraagd om hun huidige praktijkperiode te evalueren. Wanneer de aios ten tijde van het onderzoek een externe stage liep, werd deze verzocht om de vragen voor de meest recente praktijkperiode van het eigen profiel in te vullen. Verder hebben we de contactpersonen van de 39 praktijkopleidingsinstellingen gevraagd om onze vragenlijst door te sturen naar alle andere artsen binnen de praktijkopleidingsinstelling die betrokken zijn bij het opleiden van aios. De reacties van artsen van die laatsten en die van de praktijkopleiders werden gegroepeerd, omdat ze allemaal het perspectief van de begeleider vertegenwoordigen. Beide groepen worden onder de term supervisor geschaard.

De vragenlijsten werden ingevuld via een online platform (SurveyMonkey). Tot drie keer toe is er een automatische herinnering per e‑mail verstuurd naar non-responders. De gegevensverzameling duurde een maand.

### Vragenlijst

We gebruikten de Dutch Residency Educational Climate Test (D-RECT) als basis om ons onderzoek uit te voeren [23, 24]. De D‑RECT is een gevalideerd instrument om het leerklimaat voor aios te evalueren en is ontwikkeld in het Nederlands. Het bestaat uit 35 items met een negenfactorenstructuur: opleidingssfeer, werken in een team, rol van de formele opleider, begeleiden en toetsen, gepland onderwijs, samenwerking met peers, aansluiten van het werk bij de aios, toegankelijkheid van supervisors en overdracht. Items worden gescoord op een vijfpuntslikertschaal (1 = helemaal mee oneens, 2 = niet mee eens, 3 = neutraal, 4 = mee eens, 5 = helemaal mee eens) en kunnen ook worden beantwoord met ‘niet van toepassing’. Drie aios-evaluaties per afdeling zijn nodig om het algehele leerklimaat betrouwbaar te beoordelen en acht aios-evaluaties zijn nodig om de subschalen te beoordelen [24]. Na overleg met instituutopleiders van de NSPOH en TNO hebben we enkele vragen van de D‑RECT aangepast aan het opleidingssysteem en het werkveld van de arts M + G. We hebben de subschaal Gepland onderwijs verwijderd, omdat cursorisch onderwijs niet plaatsvindt binnen de opleidingsinstelling, maar op een centraal niveau wordt georganiseerd door de NSPOH of TNO en afzonderlijk wordt beoordeeld. Ook hebben we de subschaal Overdracht verwijderd omdat deze niet van toepassing is op de werkomgeving van de aios M + G. We hebben vragen toegevoegd om aspecten te evalueren die specifiek zijn voor de werkomgeving van de aios M + G. De aangepaste lijst is beschikbaar als Bijlage 1 (zie digitaal aanvullende content). Om een supervisorversie van de D‑RECT te maken, hebben we de vragen van aios ‘vertaald’ naar supervisors. Bijvoorbeeld aios vraag 1 *Als ik een supervisor nodig heb, kan ik er altijd een bereiken* werd veranderd in een supervisorvraag *Als een aios een supervisor nodig heeft, kan hij/zij er altijd een bereiken.*

### Data-analyse

Met beschrijvende statistieken werden de kenmerken van de onderzoekspopulatie samengevat. Statistische analyses werden uitgevoerd in SPSS-28.

Ten tweede werd de interne consistentie van de subschalen met behulp van Cronbach’s α geschat, zowel voor de vragenlijst van de aios als voor die van de supervisor. In het algemeen wordt een α‑waarde van 0,70 of meer als bevredigend beschouwd. Aangepaste items werden getest op hun bijdrage aan Cronbach’s α. Als Cronbach’s α met 0,02 of meer verbeterde door het aangepaste item te verwijderen, hebben we dit item niet opgenomen in de subschaal en afzonderlijk gerapporteerd.

Ten derde werden de gemiddelde uitkomstscores berekend voor elke D‑RECT-subschaal door de totale subschaalscore te delen door het aantal subschaalvragen. De totale gemiddelde D‑RECT-score werd berekend als het gemiddelde van alle itemscores gedeeld door het totale aantal vragen. Gemiddelde scores werden voor aios en supervisors afzonderlijk berekend en opgesplitst per profiel. Scores boven de 4 duiden op een positieve houding ten opzichte van de beoordeelde subschaal.

Ten slotte werden de antwoorden van aios en supervisors per profiel op het niveau van opleidingsinstelling gematcht om de beleving van aios en supervisors te kunnen vergelijken. Subschaalgemiddelden en itemscores van de aios werden afgetrokken van de subschaalgemiddelden van hun gematchte supervisor. Het gemiddelde van het verschil tussen aios en supervisors werd berekend. Reacties van aios en supervisors die niet konden worden gematcht, zijn buiten deze analyse gelaten. Subschaalverschillen van 0,6 of hoger werden als opmerkelijk beschouwd.

### Ethische goedkeuring

Ethische goedkeuring is ontvangen van de NVMO-Ethische Toetsingscommissie, protocolnummer 2021.3.4. De deelnemers werd verteld dat anonimiteit gegarandeerd was en dat deelname vrijwillig was. Informed consent werd verkregen.

## Resultaten

In totaal hebben 114 van de 224 aios M + G die in 2019 en later met hun opleiding zijn begonnen gereageerd (totaal responspercentage 50,9 %, per profiel: 51,8 % JGZ, 59,3 % ITM, 33,3 % FG). Van de JGZ- aios was 93 % vrouwelijk, voor het ITM- en FG-profiel was dit respectievelijk 78,1 % en 54,6 %.

Cronbach’s α-test resulteerde in het verwijderen van één toegevoegde vraag uit de D‑RECT-subschaal Toegankelijkheid supervisors (vraag 5) en één toegevoegde vraag uit de subschaal Begeleiden en toetsen (vraag 11).

De uitkomstscores zijn samengevat in tab. 1, met daarin de gemiddelden en standaarddeviaties voor de zeven subschalen per profiel. De gemiddelde subschaalscores waren op alle subschalen hoger dan 4,0, behalve op de schaal Samenwerking peers (gemiddelde = 3,65). Inzoomend op vraagniveau bleek dat samenwerking binnen het eigen profiel beoordeeld werd met een score van 4,17. Samenwerking tussen aios van verschillende profielen scoorde echter slechts 2,73. Dit verschil tussen samenwerking binnen en tussen profielen zagen we in alle drie de profielen (JGZ 3,91 versus 2,46, ITM 4,71 versus 3,45, FG 4,44 versus 2,67). De gemiddelde scores voor de aios in het FG-profiel zaten voor de meeste schalen onder het gemiddelde, alleen de subschaal Toegankelijkheid van supervisors had een score boven de 4,0.

**Table 1:** 

	M + G totaal	JGZ	ITM	FG
	*n* = 114	*n* = 71	*n* = 32	*n* = 11
	gemiddelde (sd)	gemiddelde (sd)	gemiddelde (sd)	gemiddelde (sd)
subschaal Toegankelijkheid supervisors	4,65 (0,42)	4,67 (0,42)	4,70 (0,35)	4,41 (0,58)
subschaal Begeleiden en toetsen	4,09 (0,67)	4,17 (0,57)	4,03 (0,77)	3,67 (0,85)
subschaal Werken in een team	4,31 (0,55)	4,32 (0,54)	4,44 (0,48)	3,90 (0,67)
subschaal Samenwerking peers^a^	3,65 (0,98)	3,40 (1,02)	4,17 (0,76)	3,83 (0,61)
subschaal Opleidingssfeer	4,44 (0,74)	4,66 (0,47)	4,34 (0,66)	3,38 (1,33)
subschaal Rol formele opleider	4,24 (0,55)	4,30 (0,46)	4,27 (0,52)	3,79 (0,94)
subschaal Aansluiten werk bij aios	4,05 (0,55)	4,08 (0,54)	4,00 (0,56)	3,96 (0,61)
Totale score	4,19 (0,43)	4,25 (0,35)	4,21 (0,42)	3,76 (0,68)

^a^N voor de subschaal Samenwerken peers = 107, 68, 30 en 9 voor respectievelijk M + G totaal, JGZ, ITM en FG

In totaal ontvingen we reacties van 106 supervisors van 36 opleidingsinstellingen. Voor 78,8 % (26/33) van de opleidingsinstellingen die het profiel JGZ aanbieden hebben we een match kunnen vormen tussen aios en supervisor. Een match werd gevormd voor 38,1 % (8/21) van de opleidingsinstellingen die het ITM-profiel aanbieden en voor 33,3 % (5/15) die het FG-profiel aanbieden. De gemiddelde verschillen tussen supervisors en aios waren over het algemeen minder dan 0,6, waarbij positieve scores erop wezen dat de supervisors de subschaal iets hoger beoordeelden dan de aios (tab. 2). Alleen in het profiel ITM voor de subschaal Begeleiden en toetsen en in het profiel FG voor de subschalen Samenwerking peers en Opleidingssfeer was het verschil 0,6 of meer.

**Table 2:** 

	M + G totaal	JGZ	ITM	FG
	*n*^a^ = 39	*n*^a^ = 26	*n*^a^ = 8	*n*^a^ = 5
	gemiddelde (sd)	gemiddelde (sd)	gemiddelde (sd)	gemiddelde (sd)
subschaal Toegankelijkheid supervisors	0,07 (0,44)	0,04 (0,46)	0,1 (0,37)	0,2 (0,48)
subschaal Begeleiden en toetsen	0,40 (0,59)	0,31 (0,57)	0,61 (0,49)	0,58 (0,8)
subschaal Werken in een team	−0,03 (0,65)	−0,22 (0,59)	0,31 (0,46)	0,46 (0,8)
subschaal Samenwerken peers^b^	0,29 (1,36)	0,33 (1,38)	−0,09 (1,37)	1 (1,32)
subschaal Opleidingssfeer	0,18 (0,64)	0,06 (0,48)	0,29 (0,63)	0,67 (1,16)
subschaal Rol formele opleider	0,24 (0,45)	0,18 (0,4)	0,41 (0,39)	0,25 (0,78)
subschaal Aansluiten werk bij aios	0,17 (0,55)	0,12 (0,57)	0,29 (0,37)	0,21 (0,75)
Totale score	0,19 (0,43)	0,11 (0,4)	0,32 (0,23)	0,4 (0,73)

^a^*n* = aantal supervisor-aios-matches

^b^Subschaal Samenwerken peers *n* = 37 voor M + G totaal en *n* = 3 voor FG

## Beschouwing

### Voornaamste bevindingen

De algemene beoordeling van het leerklimaat door aios M + G was positief, met een gemiddelde score van 4,19 op een vijfpuntsschaal. Samenwerking tussen aios oftewel Samenwerking peers scoorde het minst gunstig, vanwege de lage scores op samenwerking tussen profielen. Er waren geen noemenswaardige verschillen in de beleving van aios en supervisors met betrekking tot het leerklimaat.

### Verklaringen van de bevindingen

#### Leerklimaat

Nederlandse aios M + G ervaren het leerklimaat op landelijk niveau als positief. De lagere totale gemiddelde score en subschaalscores voor het profiel FG, vergeleken met de scores van alle aios M + G, moeten voorzichtig worden geïnterpreteerd vanwege het kleine aantal aios profiel FG en de relatief lagere respons in dit profiel. Een bijkomend aspect is dat de FG-opleiding een grotere verandering heeft ondergaan dan de andere profielen en volledig is vernieuwd van een cursus van enkele maanden naar een tweejarige opleiding. Het is aannemelijk dat de FG-opleidingsinstellingen door deze curriculumverandering voor een grotere uitdaging hebben gestaan dan de andere profielen, waar de curriculumveranderingen minder ingrijpend waren.

M + G-scores voor de subschaal Samenwerking peers waren slecht in vergelijking met de andere subschalen. Dit bleek het gevolg van slechte scores voor het aspect samenwerking tussen aios van verschillende profielen, terwijl de samenwerking binnen het profiel beter werd beoordeeld. Om de aios M + G voor te bereiden op de praktijk van de eenentwintigste eeuw is in het vernieuwde curriculum een van de doelen het stimuleren van samenwerking tussen alle profielen arts M + G [25]. Maatschappelijke veranderingen en de toename van chronische aandoeningen vragen om een versterking van de preventieve zorg, waar meer samenwerking tussen de verschillende M + G-profielen een belangrijke bijdrage aan kan leveren. De COVID-19-pandemie laat zien hoe belangrijk samenwerking is met en tussen professionals in de publieke gezondheid op nationaal en internationaal niveau.

#### Perceptieverschillen tussen aios en supervisor

Volgens onze bevindingen lijken er geen grote verschillen te zijn in de beleving van het leerklimaat tussen aios M + G en hun supervisors. Voor een aantal subschalen in de profielen ITM en FG werden echter wel verschillen gevonden tussen de beleving van de aios en die van de supervisor, maar vanwege het kleine aantal aios-supervisor-matches voor deze profielen kunnen hieruit geen harde conclusies worden getrokken.

Onderzoek onder aios chirurgie en hun supervisors naar aspecten die onderdeel zijn van het leerklimaat, zoals onderwijsprestaties en feedback [26, 27], lieten een slechte correlatie zien tussen de evaluatie van aios en supervisors. Dit verschil met onze uitkomsten kan worden verklaard door de verschillen tussen de werksituatie van de arts M + G en die van de chirurg. Een andere verklaring zou kunnen zijn dat supervisors in ons onderzoek het leerklimaat binnen de opleidingsinstelling in het algemeen moesten beoordelen, terwijl in de andere onderzoeken supervisors hun eigen rol moesten evalueren met vragenlijsten voor zelfevaluatie.

### Sterke punten en beperkingen

Voor zover ons bekend is dit het eerste onderzoek dat het leerklimaat in de medische vervolgopleiding arts M + G evalueert en daarbij de percepties van aios en supervisors van het leerklimaat van de aios M + G vergelijkt. Een kracht van ons onderzoek is de landelijke opzet, waarbij alle aios M + G en hun supervisors zijn uitgenodigd om deel te nemen aan het onderzoek. Een ander sterk punt is ons behoorlijke responspercentage van ongeveer 50 % voor aios en een match tussen aios en supervisor voor 78 % van de opleidingsinstellingen voor het JGZ-profiel. Het feit dat we ondanks aanpassingen aan de originele gevalideerde D‑RECT-vragenlijst betrouwbare Cronbach’s α-waarden hebben gevonden, versterkt de waarde van onze resultaten.

Een beperking van dit onderzoek was het aantal aios en supervisors ITM en FG dat per opleidingsinstelling beschikbaar was. Daarnaast zijn door het beperkte aantal aios in opleiding tot arts M + G, niet alle supervisors van iedere opleidingsperiode aan een aios gekoppeld. Daardoor was het percentage supervisor-aios-matches voor ITM- en FG-profielen laag, en konden we voor de meeste opleidingsinstellingen het geadviseerde minimum van drie aios of supervisors niet bereiken. Dit kan van invloed zijn geweest op de betrouwbaarheid van de gevonden verschillen tussen aios en supervisors. Het ontbreken van verschillen in perceptie kan ook worden veroorzaakt door het gebruikte instrument. Een vijfpuntslikertschaal geeft een beperkte mogelijkheid om onderscheid te maken tussen niveaus van overeenstemming. Dit maakt het moeilijker om kleine verschillen in perceptie van het leerklimaat te detecteren [28].

### Praktische implicaties en toekomstig onderzoek

Dit onderzoek laat zien dat de D‑RECT-vragenlijst na kleine aanpassingen geschikt is voor het beoordelen van het leerklimaat van de medische vervolgopleiding arts M + G, met betrouwbare Cronbach’s α-waarden voor zowel de vragenlijst voor aios als die voor supervisors. Vanwege de kleine aantallen per opleidingsinstelling moeten de uitkomsten worden gezien als een eerste indicatie van de perceptie van het leerklimaat.

Het onderzoeken van gepercipieerde verschillen tussen aios en supervisors is relevant, omdat het richting geeft aan het verbeteren van het leerklimaat. Bij duidelijke verschillen in beleving moeten de supervisors om te beginnen inzicht krijgen in de behoeften van de aios en is het interessant om te achterhalen waarom supervisors het leerklimaat anders ervaren. Wanneer aios en supervisors het leerklimaat gelijkelijk beoordelen, kan de aandacht direct worden gericht op de verbeterpunten. In dit artikel blijkt dat het leerklimaat binnen de opleiding arts M + G landelijk als positief wordt ervaren, maar ook dat er wel lokale verschillen kunnen zijn die aandacht behoeven.

Om aandachtspunten met betrekking tot werkplaatsleren in de publieke gezondheid in meer detail te onderzoeken, is het aan te raden om alle relevante actoren erbij te betrekken. Hiervoor zijn naast de aios en de supervisors de volgende actoren van belang: instituutopleiders, directeuren van de opleidingsinstellingen, lokale teamleiders en opleidingsmanagers verantwoordelijk voor de implementatie van de opleiding. De aios, supervisors en instituutopleiders worden door middel van kwalitatieve interviews verdiepend gevraagd naar verbeterpunten van de nieuwe opleiding. De directeuren en managers zijn behalve voor de bedrijfsvoering ook verantwoordelijk voor het implementeren van de opleiding op lokaal niveau. Om de samenwerking tussen de verschillende profielen te bevorderen zijn onderwijskundige middelen zoals profieloverstijgende opdrachten nodig. Daarnaast is de ondersteuning op bedrijfsvoeringsniveau voor profieloverstijgend samenwerken een cruciale factor. De invloed van de bedrijfsvoering en mogelijk conflicterende bestuurskundige belangen op de opleiding arts M + G is onvoldoende bekend. GGD-directeuren en -managers worden betrokken bij vervolgonderzoek naar deze aspecten.

De vragenlijst kan verder aangepast worden aan de specifieke werk- en leeromgeving van de arts M + G. Andere kwaliteitsdomeinen, zoals beschreven in de kwaliteitsrichtlijnen van de Koepel Artsen Maatschappij + Gezondheid [22], kunnen in de toekomst in de vragenlijst worden opgenomen, waarna de vragenlijst idealiter gevalideerd zal worden. Onderwerpen die meer aandacht behoeven zijn de samenhang tussen cursorisch onderwijs en werkplekleren bij de opleidingsinstellingen, de samenwerking tussen de verschillende profielen M + G en het sociaal domein, en ten slotte het effect van supervisie op afstand op de kwaliteit van het onderwijs en leerklimaat. Dit laatste onderwerp is belangrijk, omdat uit onderzoek blijkt dat werkplekervaringen door aios in aanwezigheid van andere aios en supervisors beter als leermomenten worden herkend dan tijdens individuele werkervaringen [13].

Ons onderzoek wijst op een positief leerklimaat voor aios M + G. Omdat het onderwijs altijd evolueert en verandert, is toekomstig onderzoek nodig om het leerklimaat in de loop van de tijd te blijven evalueren.

Herhaalde evaluaties zijn ook nodig om voldoende gegevens te verzamelen om conclusies te trekken over het leerklimaat op het niveau van opleidingsinstellingen, vooral voor de kleinere profielen ITM en FG. Om de respons te verhogen wordt voor de aios een link naar de anonieme vragenlijst aan het portfolio toegevoegd. Supervisors worden jaarlijks via e‑mail benaderd. Het belang van het invullen van de vragenlijst voor de tweejaarlijkse KOERS-kwaliteitscyclus van de opleidingsinstelling wordt in de correspondentie benadrukt.

## Conclusie

Aios M + G beoordelen het leerklimaat van de opleidingsinstelling als positief en aios en supervisors lijken het leerklimaat niet anders te ervaren. Samenwerking van aios tussen profielen behoeft nader onderzoek voordat duidelijk is of verbetering nodig is en hoe werkplaatsleren daarin ondersteuning kan bieden. Onze aangepaste versie van de D‑RECT-vragenlijst lijkt geschikt om het leerklimaat binnen de medische vervolgopleiding arts M + G te evalueren, maar vervolgonderzoek is nodig om onze vragenlijst te valideren en onze bevindingen te bevestigen.
