# Supplementary material for: Resident and supervisor perception of learning climate in public health resident training in the Netherlands
Source: TSG. 2022 Nov 8;100(4):163–9. [Article in Dutch] doi: 10.1007/s12508-022-00367-6 (PMC9643892; doi:10.1007/s12508-022-00367-6)
Supplement: Supplementary file 1 [file 12508_2022_367_MOESM1_ESM.docx]

| 5 puntenschaal : | helemaal oneens | redelijk  oneens | neutraal | redelijk  eens | helemaal eens | niet van  toepassing |
| --- | --- | --- | --- | --- | --- | --- |
|  | 1 | 2 | 3 | 4 | 5 |  |

[Groene vragen komen uit de gevalideerde D-RECT, rode woorden/vragen stonden in de gevalideerde D-RECT maar zijn geschrapt omdat die niet aansluiten bij het werkveld van de arts M+G en zwarte woorden/vragen zijn toegevoegd voor M+G opleiding]

**Toegankelijkheid supervisoren Supervisie:**

Supervisor hoeft niet altijd de praktijkopleider te zijn.

1. Als ik een supervisor nodig heb, dan kan ik er altijd een bereiken.
2. Als ik wil overleggen, zijn mijn supervisoren laagdrempelig benaderbaar.
3. Het is duidelijk wie mijn werk superviseert.
4. De mate van supervisie is aangepast aan mijn ervaringsniveau.
5. Ik krijg MINIMAAL het landelijk afgesproken aantal uren supervisie per week.

**Begeleiden en toetsen:**

Supervisor hoeft niet altijd de praktijkopleider te zijn.

1. Mijn supervisoren vertellen mij uit zichzelf hoe ik functioneer.
2. De supervisoren nemen zelf het initiatief om moeilijke situaties achteraf met mij na te bespreken.
3. Mijn supervisoren toetsen of de patiëntzorg cliënt gerelateerde en/of cliënt overstijgende werkzaamheden die ik lever past verricht bij mijn niveau van bekwaamheid passen.
4. Mijn supervisoren observeren som een anamneses met een patiënt
5. Mijn supervisoren toetsen niet alleen mijn medische vakinhoudelijke expertise maar ook andere competenties zoals samenwerken, organiseren of professioneel gedrag.
6. Supervisoren geven in feedback regelmatig aan wat ik goed deed én wat ik kan verbeteren.
7. De toetsinstrumenten (KPB, PO, MSF, etc.) zijn ondersteunend bij het behalen van mijn leerdoelen.

**Werken in een team**

1. Supervisoren, verpleegkundigen, ander (paramedisch) personeel en A(N)IOS vormen hier een team.
2. Verpleegkundigen en andere zorgverleners teamleden dragen positief bij aan mijn opleiding.
3. Verpleegkundigen en andere paramedici teamleden staan open voor gezamenlijke reflectie op de gegeven patiëntenzorg werkzaamheden van het team.
4. Wanneer een dienst moet worden geruild of overgenomen, verloopt dat binnen onze A(N)IOS-groep het team zonder problemen. (vraag verplaatst van subschaal Samenwerking peers, naar subschaal Werken in een team omdat diensten ruilen binnen team en niet alleen onder A(N)IOS gebeurt)
5. Samenwerking met collega’s van andere praktijkinstellingen draagt positief bij aan mijn opleiding.

**Samenwerking peers** **binnen praktijkinstelling**

1. Er is een goede samenwerking tussen de A(N)IOS binnen mijn eigen profiel.
2. Als A(N)IOS groep zorgen we dat het werk van die dag samen gedaan wordt
3. Er is een goede samenwerking tussen de A(N)IOS van de verschillende M+G profielen.
4. Vraag verplaatst naar kopje Samenwerken in een team

**Opleidingssfeer**

1. De continuïteit in beleid wordt NIET beïnvloed door conflicten tussen supervisoren.
2. Verschil van mening in beleid tussen supervisoren wordt zó besproken dat het leerzaam is voor toehoorders.
3. Er zijn GEEN zodanig grote conflicten dat de werksfeer en daarmee de opleidingssfeer negatief beïnvloed wordt.
4. Er is (zijn) GEEN supervisor(en) die een negatieve stempel op de opleidingssfeer drukken.
5. Ik word op een correcte manier bejegend door mijn supervisoren.
6. Ik word op een correcte manier bejegend door andere teamleden.

**Rol formele praktijkopleider**

1. Mijn formele praktijkopleider weet hoever ik ben in mijn opleiding.
2. Naast de formele praktijkopleider zijn andere artsen (opleidersgroep) uit de organisatie actief betrokken bij mijn opleiding.
3. Mijn formele praktijkopleider stuurt andere leden van de opleidergroep zo nodig bij.
4. Mijn formele praktijkopleider zet zich actief in voor de kwaliteit van de opleiding.
5. Het voortgangsgesprek dat ik hier meemaak, is een zinnig gesprek over mijn functioneren.
6. Bij het voortgangsgesprek wordt stilgestaan bij mijn toekomstplannen.
7. Bij het voortgangsgesprek wordt de inbreng van meerdere supervisoren gebruikt.

**Aansluiting werk bij AIOS**

1. Het werk wat ik doe sluit aan bij mijn ervaringsniveau.
2. Het werk wat ik doe sluit aan bij wat ik op dit moment in mijn opleiding wil leren.
3. Ik krijg de mogelijkheid om voldoende tijd te besteden aan wat ik op dit moment in mijn opleiding wil leren.
4. Ik krijg de mogelijkheid patiënten om casuïstiek te vervolgen.
5. Ik krijg de mogelijkheid om actief te zijn op contextuele en cliënt-overstijgende niveaus (o.a. ‘staftaken’).
6. Het cursorisch onderwijs is ondersteunend in de praktijkopleiding.
7. Mijn formele praktijkopleider betrekt het cursorisch onderwijs bij het praktijkleren.

**Gepland onderwijs**

AIOS kunnen doorgaans bij geplande onderwijsmomenten zijn

Geplande onderwijsmomenten gaan door

Supervisoren dragen actief bij aan het tot stand komen van goed onderwijs

Het onderwijs sluit goed aan bij mijn behoefte

**Overdracht**

Supervisoren nodigen A(N)IOS uit deel te nemen aan discussies tijdens de overdracht

De overdracht wordt gebruikt als een opleidingsmoment
